# Reversible Masking Using Low-Molecular-Weight Neutral Lipids to Achieve Optimal-Targeted Delivery

**DOI:** 10.1155/2012/173465

**Published:** 2012-05-10

**Authors:** Nancy Smyth Templeton, Neil Senzer

**Affiliations:** ^1^Delivery Systems, Gradalis Inc., 2545 Golden Bear Drive, Suite 110, Carrollton, TX 75006-2317, USA; ^2^Mary Crowley Cancer Research Centers, 1700 Pacific Avenue, Suite 1100, Dallas, TX 75201, USA

## Abstract

Intravenous injection of therapeutics is required to effectively treat or cure metastatic cancer, certain cardiovascular diseases, and other acquired or inherited diseases. Using this route of delivery allows potential uptake in all disease targets that are accessed by the bloodstream. However, normal tissues and organs also have the potential for uptake of therapeutic agents. Therefore, investigators have used targeted delivery to attempt delivery solely to the target cells; however, use of ligands on the surface of delivery vehicles to target specific cell surface receptors is not sufficient to avoid nonspecific uptake. PEGylation has been used for decades to try to avoid nonspecific uptake but suffers from many problems known as “The PEGylation Dilemma.” We have solved this dilemma by replacing PEGylation with reversible masking using low-molecular-weight neutral lipids in order to achieve optimal-targeted delivery solely to target cells. Our paper will focus on this topic.

## 1. Introduction

We use bilamellar-invaginated vesicles (BIVs) that are unique liposomal nanoparticles (NPs) providing highly efficient delivery for intravenous (iv) injection of encapsulated therapeutics including plasmid DNA [1–5]. In addition to having extended half-life and stability in circulation, BIVs are nontoxic, nonimmunogenic, biodegradable and can be repeatedly administered without losing potency. Furthermore, BIVs encapsulating therapeutic agents can be modified to specifically target entry into the disease cell using small molecules that mimic beta turns incorporated on the surface of BIV complexes while bypassing nonspecific uptake using reversible masking. Although BIV-DNA complexes have already been used successfully in clinical trials to treat metastatic lung cancer [6] and hereditary inclusion body myopathy [7, 8], in the former a first-pass uptake in the involved lungs obviated the need for differential tumor targeting and, in the latter, the goal of increased production of sialic acid required merely an organ repository. However, reversible masking was designed to be used with BIVs as well as other delivery systems to focus target-specific biodistribution, for example, metastatic cancer, by bypassing nonspecific uptake post-iv injections. The aim of this review is to define and distinguish our novel reversible masking versus PEGylation and demonstrate its superior use for avoiding nonspecific uptake in vivo.

## 2. Optimization of Cationic Liposome Formulations for Use In Vivo

Much research has been directed toward the synthesis of new cationic lipids. Some new formulations enable more efficient transfection of cells in culture. However, their efficiency measured in vitro did not correlate with their ability to deliver DNA after administration in animals. Functional properties defined in vitro do not assess the stability of the complexes in plasma or their pharmacokinetics and biodistribution, all of which are essential for optimal activity in vivo. Colloidal properties of the complexes, in addition to the physicochemical properties of their component lipids, also determine these parameters. In particular, in addition to efficient transfection of target cells, nucleic acid-liposome complexes must be able to traverse tight barriers in vivo and penetrate throughout the target tissue to produce efficacy for the treatment of disease, that is, countercurrent to increased intratumoral pressure gradients for the treatment of cancer. These are not issues for achieving efficient transfection of cells in culture with the exception of polarized tissue culture cells. Therefore, we are not surprised that optimized liposomal delivery vehicles for use in vivo may be different than those used for efficient delivery to some cells in culture.

 In summary, in vivo nucleic acid-liposome complexes that produce efficacy in animal models of disease have extended half-life in the circulation, are stable in serum, have broad biodistribution that can be focused, efficiently encapsulate various sizes of nucleic acids, are targetable to specific organs and cell types, penetrate across tight barriers in several organs, penetrate evenly throughout the target tissue, are optimized for nucleic acid : lipid ratio and colloidal suspension in vivo, can be size fractionated to produce a homogenous population of complexes prior to injection, and can be repeatedly administered. Recently, we demonstrated efficacy of a robust liposomal delivery system in small and large animal models for lung [18], breast [19], head and neck, and pancreatic cancers [20–22], and for Hepatitis B and C [23]. Based on efficacy in these animal studies, this liposomal delivery system has been used successfully in phase I clinical trials to treat end-stage nonsmall cell lung carcinoma patients who have failed to respond to chemotherapy [6] and hereditary inclusion body myopathy [7, 8]. The nonsmall cell lung carcinoma patients have prolonged life spans and have demonstrated objective responses including tumor regression. Efficacy was also demonstrated for the single patient trials for hereditary inclusion body myopathy. The BIV delivery system will also be used in upcoming clinical trials to treat other types of cancer including pancreatic, breast, and head and neck cancers. Our studies have demonstrated broad efficacy in the use of liposomes to treat disease and have dispelled several myths that exist concerning the use of liposomal systems.

## 3. Liposome Morphology and Effects on Gene Delivery and Expression

**Figure 1:**
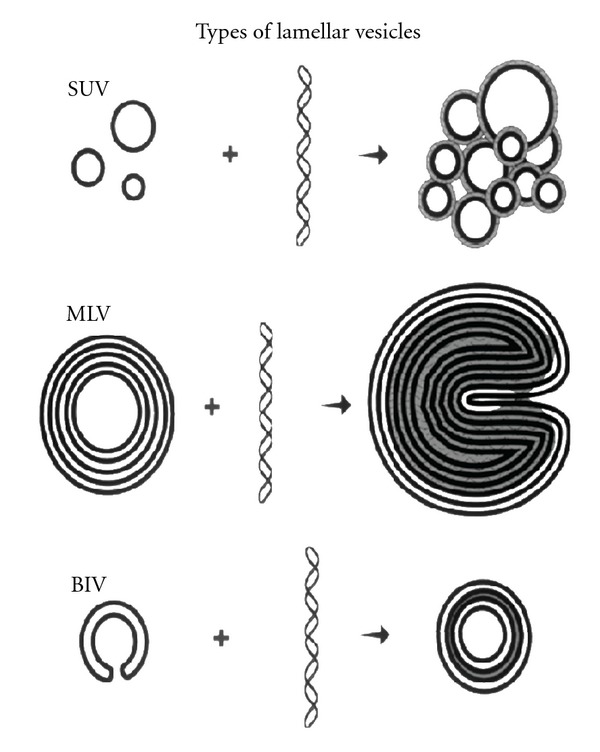
Diagrams drawn from cryo-electron micrographs of cross-sections through vitrified films of various types of liposomes and DNA-liposome complexes. SUVs are small unilamellar vesicles that condense nucleic acids on the surface and produce “spaghetti and meatballs” structures. MLVs are multilamellar vesicles that appear as “Swiss rolls” after mixing with DNA. BIVs are bilamellar-invaginated vesicles produced using a formulation developed in our laboratory [1]. Nucleic acids are efficiently encapsulated between two bilamellar-invaginated structures (BIVs).

Efficient in vivo nucleic acid-liposome complexes have unique features including their morphology, mechanisms for crossing the cell membrane and entry into the nucleus, ability to be targeted for delivery to specific cell surface receptors, and ability to penetrate across tight barriers and throughout target tissues. Liposomes have different morphologies based upon their composition and the formulation method. Furthermore, the morphology of complexes can contribute to their ability to deliver nucleic acids in vivo. Formulations frequently used for the delivery of nucleic acids are lamellar structures including small unilamellar vesicles (SUVs), multilamellar vesicles (MLVs), or the bilamellar invaginated vesicles (BIVs) recently developed in our laboratory Figure 1. Several investigators have developed liposomal delivery systems using hexagonal structures; however, they have demonstrated efficiency primarily for the transfection of some cell types in culture and not for in vivo delivery. SUVs condense nucleic acids on the surface and form “spaghetti and meatballs” structures [24]. DNA-liposome complexes made using SUVs produce little or no gene expression upon systemic delivery, although these complexes transfect numerous cell types efficiently in vitro [25, 26]. Furthermore, SUV liposome-DNA complexes cannot be targeted efficiently. SUV liposome-DNA complexes also have a short half-life within the circulation, generally about 5 to 10 minutes. Polyethylene glycol (PEG) has been added to liposome formulations to extend their half-life [27–29]; however, PEGylation creates other problems that have not as yet been resolved. PEG seems to hinder delivery of cationic liposomes into cells due to its sterically hindering ionic interactions, and it interferes with optimal condensation of nucleic acids onto the cationic delivery vehicle. Furthermore, the resultant extremely long half-life in the circulation, for example, up to several days, has caused problems for patients as illustrated by the increased percentage of injected dose of the PEGylated liposomal formulation doxil that encapsulates the cytotoxic agent, doxorubicin, which accumulates in the skin, hands, and feet resulting in mucositis and hand and foot syndrome [30, 31] that cause extreme discomfort to the patient. Attempts to add ligands to doxil for delivery to specific cell surface receptors have not resulted in much cell-specific delivery, and an increased percentage of the injected targeted formulation still accumulates in the skin, hands, and feet. Addition of PEG into formulations developed in our laboratory also caused steric hindrance in the bilamellar-invaginated structures that hindered DNA encapsulation, and gene expression was substantially diminished. Recent efforts to use cleavable PEG are unimpressive and have not solved these problems [10, 12–17, 32]. The vast majority of the injected PEGylated complexes bypass the target cell, including those using cleavable PEG.

Some investigators have loaded nucleic acids into SUVs using a variety of methods; however, the bulk of the DNA does not load or stay within the liposomes. Furthermore, most of the processes used for loading nucleic acids within liposomes are extremely time-consuming and not cost-effective. Therefore, SUVs are not the ideal liposomes for creating nonviral vehicles for targeted delivery.

Complexes made using MLVs appear as “Swiss rolls” when viewing cross-sections by cryo-electron microscopy [33]. These complexes can become too large for systemic administration or deliver nucleic acids inefficiently into cells due to inability to “unravel” at the cell surface. Addition of ligands onto MLV liposome-DNA complexes further aggravates these problems. Therefore, MLVs are not useful for the development of targeted delivery of nucleic acids.

Using a formulation developed in our laboratory, nucleic acids are efficiently encapsulated between two bilamellar invaginated vesicles, BIVs [1]. We created these unique structures using 1,2-bis(oleoyloxy)-3-(trimethylammino)propane (DOTAP) and synthetic cholesterol (Chol) and a novel formulation procedure. This procedure is different because it includes a brief, low-frequency sonication followed by manual extrusion through filters of decreasing pore size. The 0.1 and 0.2 um filters used are made of aluminum oxide and not polycarbonate that is typically used by other protocols. Aluminum oxide membranes contain more pores per surface area that are evenly spaced and sized and have straight channels. During the manual extrusion process, the liposomes are passed through each of four different sized filters only once. This process produces 88% invaginated liposomes. Use of high frequency sonication and/or mechanical extrusion produces only SUVs.

**Figure 2:**
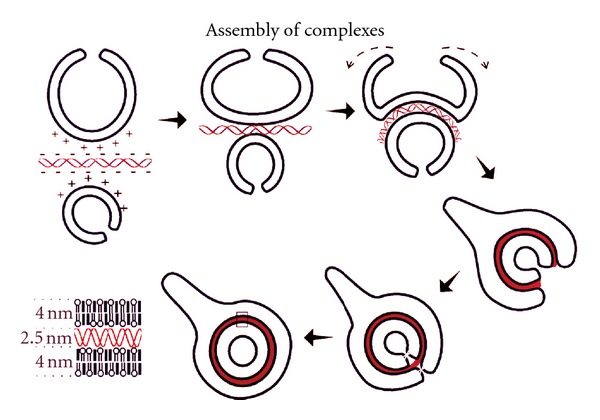
Proposed model showing cross-sections of extruded DOTAP: Chol liposomes (BIVs) interacting with nucleic acids. Nucleic acids adsorb onto a BIV via electrostatic interactions. Attraction of a second BIV to this complex results in further charge neutralization. Expanding electrostatic interactions with nucleic acids causes inversion of the larger BIV and total encapsulation of the nucleic acids. Inversion can occur in these liposomes because of their excess surface area, which allows them to accommodate the stress created by the nucleic acid-lipid interactions. Nucleic acid binding reduces the surface area of the outer leaflet of the bilayer and induces the negative curvature due to lipid ordering and reduction of charge repulsion between cationic lipid headgroups. Condensation of the internalized nucleic acid-lipid sandwich expands the space between the bilayers and may induce membrane fusion to generate the apparently closed structures. The enlarged area shows the arrangement of nucleic acids condensed between two 4 nm bilayers of extruded DOTAP:Chol.

**Figure 3:**
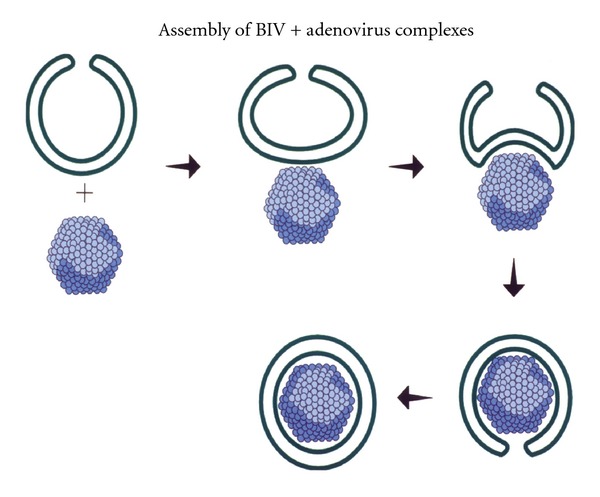
Proposed model showing cross-sections of an extruded DOTAP:Chol liposome (BIV) interacting with adenovirus. Adenovirus interacts with a BIV causing negative curvature and wrapping around the virus particle.

BIVs condense unusually large amounts of nucleic acids of any size Figure 2 as well as viruses Figure 3. Furthermore, addition of other DNA condensing agents including polymers is not necessary. For example, condensation of plasmid DNA onto polymers prior to encapsulation in the BIVs did not increase condensation or subsequent gene expression after transfection in vitro or in vivo. Encapsulation of nucleic acids by these BIVs alone is spontaneous and immediate, and, therefore, cost-effective requiring only one step of simple mixing. The extruded BIV DOTAP : Chol-nucleic acid complexes are also large enough so that they are not cleared rapidly by Kupffer cells in the liver and yet extravasate across tight barriers, including the endothelial cell barrier of the lungs in a normal mouse, and diffuse through target organs efficiently [18]. Our work demonstrating efficacy for treatment of nonsmall cell lung cancer [18] showed that only BIV DOTAP:Chol-p53 DNA liposome complexes produced efficacy; whereas SUV DOTAP : Chol-p53 DNA liposome complexes produced no efficacy. Therefore, the choice of lipids alone is not sufficient for optimal DNA delivery, and the morphology of the complexes is essential.

## 4. Optimal Lipids and Liposome Morphology: Effects on Gene Delivery and Expression

Choosing the best cationic lipids and neutral lipids are also essential for producing the optimal in vivo formulation. For example, using our novel manual extrusion procedure does not produce BIVs using the cationic lipid dimethyldioctadecylammonium bromide (DDAB). Furthermore, DOTAP is biodegradable, whereas DDAB is not biodegradable. Use of biodegradable lipids is preferred for use in humans. Furthermore, only DOTAP and not DDAB containing liposomes produced highly efficient gene expression in vivo [1]. DDAB did not produce BIVs and was unable to encapsulate nucleic acids. Apparently, DDAB and DOTAP containing SUVs produce similar efficiency of gene delivery in vivo; however, these SUVs are not as efficient as BIV DOTAP : Chol [1]. In addition, use of L-*α* dioleoyl phosphatidylethanolamine (DOPE) as a neutral lipid creates liposomes that cannot wrap or encapsulate nucleic acids. Several investigators have reported efficient transfection of cells in culture using DOPE in liposomal formulations. However, our data showed that formulations consisting of DOPE were not efficient for producing gene expression in vivo [1].

Investigators must also consider the source and lot variability of certain lipids purchased from companies. For example, different lots of natural cholesterol from the same vendor can vary dramatically and will affect the formulation of liposomes. We use synthetic cholesterol instead of natural cholesterol that is purified from the wool of sheep. Synthetic cholesterol is required by the Food and Drug Administration for use in producing therapeutics for injection into humans.

Our BIV formulations are also stable for a few years as liquid suspensions. Freeze-dried formulations can also be made that are stable indefinitely even at room temperature. Stability of liposomes and liposomal complexes is also essential particularly for the commercial development of human therapeutics.

## 5. Liposome Encapsulation, Flexibility, and Optimal Colloidal Suspensions

**Figure 4:**
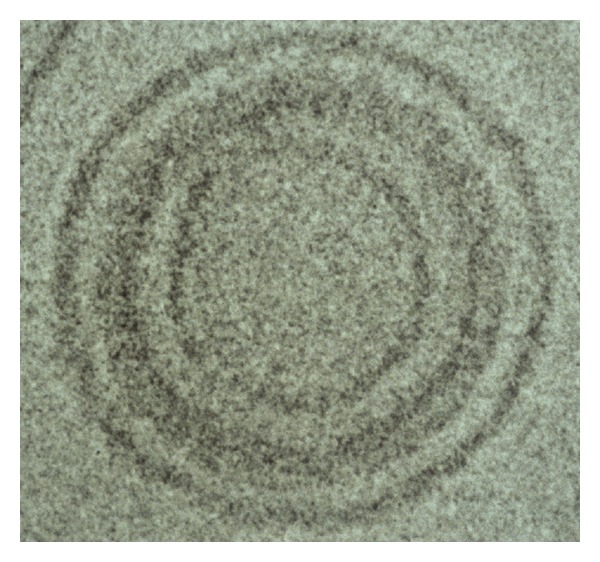
Cryo-electron micrograph of BIV DOTAP : Chol-DNA liposome complexes. The plasmid DNA is encapsulated between two BIVs.

**Figure 5:**
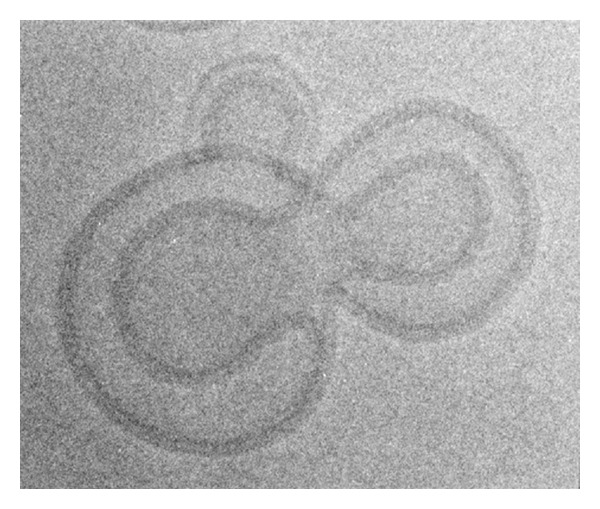
Cryo-electron micrograph of extruded DOTAP: DOPE liposomes complexed to plasmid DNA. Although these liposomes were prepared by the same protocol that produces BIV DOTAP : Chol, these vesicles cannot wrap and encapsulate nucleic acids. The DNA condenses on the surfaces of the liposomes shown.

 A common belief is that artificial vehicles must be 100 nm or smaller to be effective for systemic delivery. However, this belief is most likely true only for large, inflexible delivery vehicles. Blood cells are several microns (up to 7000 nm) in size and yet have no difficulty circulating in the blood including through the smallest capillaries. However, sickle cell blood cells, that are rigid, do have problems in the circulation. Therefore, we believe that flexibility is a more important issue than small size. In fact, BIV DNA-liposome complexes in the size range of 200 to 450 nm produced the highest levels of gene expression in all tissues after iv injection [1]. Delivery vehicles, including nonviral vectors and viruses, that are not PEGylated and are smaller than 200 nm are cleared quickly by the Kupffer cells in the liver. Therefore, increased size of liposomal complexes could extend their circulation time particularly when combined with injection of high colloidal suspensions. BIVs are able to encapsulate nucleic acids and viruses apparently due to the presence of cholesterol in the bilayer (Figure 4). Formulations including DOPE instead of cholesterol could not assemble nucleic acids by a “wrapping type” of mechanism (Figure 5) and produced little gene expression in the lungs and no expression in other tissues after intravenous injections. Because the extruded DOTAP : Chol BIV complexes are flexible and not rigid, are stable in high concentrations of serum, and have extended half-life, they do not have difficulty circulating efficiently in the bloodstream.

We believe that colloidal properties of nucleic acid-liposome complexes also determine the levels of gene expression produced after in vivo delivery [1, 34]. These properties include the DNA : lipid ratio that determines the overall charge density of the complexes and the colloidal suspension that is monitored by its turbidity. Complex size and shape, lipid composition and formulation, and encapsulation efficiency of nucleic acids by the liposomes also contribute to the colloidal properties of the complexes. The colloidal properties affect serum stability, protection from nuclease degradation, blood circulation time, and biodistribution of the complexes.

Our in vivo transfection data showed that an adequate amount of colloids in suspension was required to produce efficient gene expression in all tissues examined [1]. The colloidal suspension is assessed by measurement of adsorbance at 400 nm using a spectrophotometer optimized to measure turbidity. Our data showed that transfection efficiency in all tissues correlated with OD400 of the complexes measured prior to intravenous injection.

## 6. Efficient Dissemination throughout target Tissues and Migration across Tight Barriers

A primary goal for efficient in vivo delivery is to achieve extravasation into and penetration throughout the target organ/tissue ideally by minimally invasive systemic administration. Without these events, therapeutic efficacy is highly compromised for any treatment including gene and drug therapies. Achieving this goal is difficult due to the many tight barriers that exist in animals and people. Furthermore, many of these barriers become tighter in the transition from neonates to becoming adults. Penetration throughout an entire tumor is further hindered due to the increased interstitial pressure within most tumors [35–37]. We believe that nonviral systems can play a pivotal role in achieving target organ extravasation and penetration needed to treat or cure certain diseases. Our preliminary studies have shown that extruded BIV DOTAP : Chol nucleic acid : liposome complexes can extravasate across tight barriers and penetrate evenly throughout entire target organs, whereas viral vectors cannot cross identical barriers. As stated above, these barriers include the endothelial cell barrier in a normal mouse [18, 38], the posterior blood retinal barrier in adult mouse eyes [38], complete and homogeneous diffusion throughout large tumors [18, 38], and penetration through several tight layers of smooth muscle cells in the arteries of pigs [38]. Diffusion throughout large tumors was measured by expression of ß-galactosidase or the proapoptotic gene p53 in about half of the p53-null tumor cells after a single injection of BIV DOTAP : Chol-DNA liposome complexes into the center of a tumor. Transfected cells were evenly spread throughout the tumors. Tumors injected with complexes encapsulating plasmid DNA encoding p53 showed apoptosis in almost all of the tumor cells by TUNEL staining. Tumor cells expressing p53 mediate a bystander effect on neighboring cells perhaps due to upregulation by Fas ligand that causes nontransfected tumor cells to undergo apoptosis.

## 7. Charge versus Delivery

**Figure 6:**
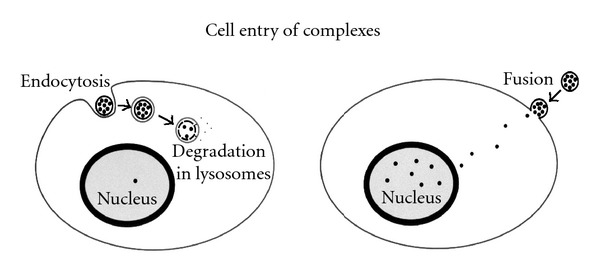
Mechanisms for cell entry of nucleic acid-liposome complexes. Two major pathways for interaction are by endocytosis or by direct fusion with the cell membrane. Complexes that enter the cell by direct fusion allow delivery of more nucleic acids to the nucleus because the bulk of the nucleic acids do not enter endosomes.

Our delivery system is efficient because we have optimized the overall charge of complexes to produce the highest delivery into cells, that is approximately 45.5 mV measured by a zeta potential analyzer [9]. Our complexes deliver DNA into cells by fusion with the cell membrane and thereby avoid the endocytic pathway (Figure 6). Cells are negatively charged on the surface, and specific cell types vary in their density of negative charge. These differences in charge density can influence the ability of cells to be transfected. Cationic complexes have nonspecific ionic charge interactions with cell surfaces. Efficient transfection of cells by cationic complexes is, in part, contributed by adequate charge interactions. In addition, other publications report that certain viruses have a partial positive charge around key subunits of viral proteins on the virus surface responsible for binding to and internalization through target cell surface receptors [39–44]. Therefore, this partial positive charge is required for virus entry into the cell. Thus, maintenance of adequate positive charge on the surface of targeted BIV complexes is essential for optimal delivery into the cell. Different formulations of liposomes interact with cell surfaces via a variety of mechanisms. Two major pathways for interaction are by endocytosis or by direct fusion with the cell membrane [33, 45–50]. Preliminary data suggest that nucleic acids delivered in vitro and in vivo using BIV complexes developed in our lab enter the cell by direct fusion. Apparently, with our delivery vehicle, the bulk of the nucleic acids do not enter endosomes, and, therefore, the bulk of nucleic acids enter the nucleus more rapidly. Fusogenic cell transfection produced orders of magnitude increased levels of gene expression and increased numbers of cells transfected versus cells transfected through the endocytic pathway.

## 8. Reversible Masking

**Figure 7:**
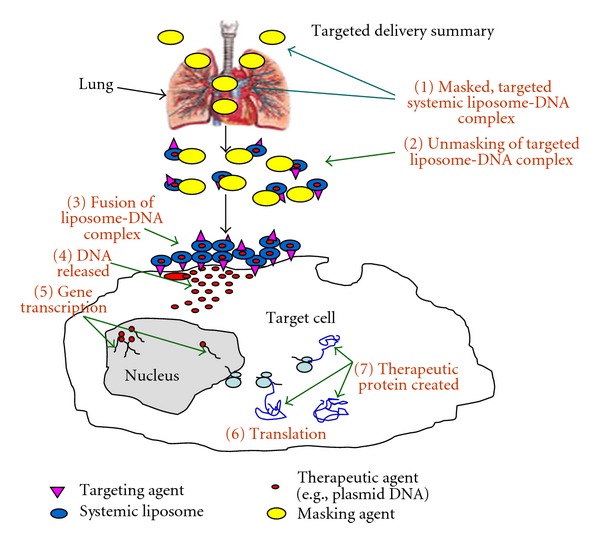
Optimized strategy for delivery and gene expression in the target cell. Optimization of many steps is required to achieve targeted delivery, shielding from nonspecific uptake in nontarget organs and tissues, deshielding, fusion with the cell membrane, entry of nucleic acids into the cell and to the nucleus, and production of gene expression of a cDNA cloned in a plasmid.

However, the positive charge on the surface of delivery vehicles also results in uptake in nontarget cells as well. Therefore, the charge must be shielded briefly until the complexes arrive at the target cell. As stated above, we believe that maintenance of adequate positive charge on the surface of complexes is essential to drive cell entry by direct fusion. Therefore, we created a methodology to achieve targeted delivery of our complexes in vivo without the use of PEG. These ligand-coated BIV complexes reexpose the overall positive charge of the complexes as they approach the target cells. In addition, through covalent attachments, we have added small molecules to the surface of our preformed complexes that mimic protein-protein interactions [9]. These small molecules efficiently bind to the target cell surface receptor and maintain entry into the cell by direct fusion. Furthermore, we showed that using this novel method of addition of ligands to the complexes for targeted delivery results in further increased gene expression in the target cells after transfection. Therefore, this design of a targeted liposomal delivery system retains predominant fusogenic cell entry rather than the endocytic transport.  Figure 7 shows our optimized strategy to achieve targeted delivery, deshielding, fusion with the cell membrane, entry of nucleic acids into the cell and to the nucleus, and production of gene expression of a cDNA cloned in a plasmid.

Much effort has been made to specifically deliver nucleic acid-liposome complexes to target organs, tissues, and/or cells. Ligands that bind to cell surface receptors are usually attached to PEG and then attached to the cationic or anionic delivery vehicle. Due to shielding of the positive charge of cationic complexes by constitutively incorporated PEG, delivery to the specific cell surface receptor can be accomplished by only a small fraction of complexes injected systemically. Furthermore, delivery of PEGylated complexes into the cell occurs predominantly through the endocytic pathway, and subsequent degradation of the bulk of the nucleic acid occurs in the lysosomes. Thus, gene expression is generally lower in the target cell than when using the nonspecific delivery of highly efficient cationic complexes. Recent efforts to use cleavable PEG are unimpressive and have not solved these problems (Table 1) [10, 12–17, 32].

As discussed above, the vast majority of the injected PEGylated complexes bypass the target cell, including those using cleavable PEG. Apparently, the PEGylated complexes cannot utilize critical charge interactions for optimal transfection into cells by direct fusion due to the overall low or neutral charge. The inability to expose positive charge on the surface of optimized delivery vehicles results in the transfection of fewer cells. PEGylation was first used to increase the half-life of complexes in the circulation and to avoid uptake in the lung. However, this technology also destroys the ability to efficiently transfect cells. We were able to increase the half-life in circulation of BIVs to five hours without the use of PEG. Because the extended half-life of BIVs is not too long, this delivery system does not result in the accumulation of complexes in nontarget tissues that occurs with circulation half-lives of one to three days as seen with PEGylated liposomal delivery systems. Some investigators have now reported targeted delivery that produces increased gene expression in the target cell over their nontargeted complexes. However, these nontargeted and targeted delivery systems are inefficient [51] compared to efficient delivery systems such as the BIVs.

In using the extruded BIV DOTAP : Chol nucleic acid : liposome complexes, we produced an optimal half-life in the circulation without the use of PEG [9]. Extended half-life was produced primarily by the formulation, preparation method, injection of optimal colloidal suspensions, serum stability, and optimal nucleic acid : lipid ratio used for mixing complexes, and size (200 to 450 nm). Furthermore, we avoid uptake in the lungs using the negative charge of the ligands and “shielding/deshielding compounds” that can be added to the complexes used for targeting just prior to injection or administration in vivo. Our strategy to bypass nonspecific transfection is called reversible masking (US Patent no. 7,037,520 B2) [9] which allows for charge reexposure facilitated by first-pass circulatory sheering forces. As discussed above, we believe that use of small molecules for targeted delivery is ideal, and smaller ligands require the use of smaller reversible masks. Therefore, we accomplished decreasing the overall charge of BIV complexes by adding the small neutral lipid, *n*-dodecyl-*β*-D-maltopyranoside, approximately 511 MW, just prior to iv injections [9](Templeton, N.S. US Patent no. 7,037,520 B2 issued May 2, 2006). By addition of ligands using the novel approaches that we developed, adequate overall positive charge on the surface of complexes at the target site is preserved. In summary, we achieve optimal circulation time of the complexes, reach and deliver to the target organ, avoid uptake in nontarget tissues, and efficiently interact with the cell surface to produce optimal transfection.

We have developed a multidisciplinary approach combining molecular biology, delivery technology, combinatorial chemistry, and reversible masking to create improved systemic, targeted delivery of plasmid DNA while avoiding nonspecific uptake in vivo. We applied this technology to efficiently target delivery to a human tumor-microenvironment model. We achieved efficient, targeted delivery by attachment of specific targeting ligands to the surface of our BIV complexes in conjunction with reversible masking to bypass nonspecific tissues and organs. We identified ligands that target a human tumor-microenvironment created *in vitro *by coculturing primary human endothelial cells with human lung or pancreatic cancer cells. The model was confirmed by increased expression of tumor endothelial phenotypes including CD31 and VEGF-A and prolonged survival of endothelial capillary-like structures. The cocultures were used for high-throughput screening of a specialized small-molecule peptidomimetic library to identify ligands specific for human tumor-associated endothelial cells *in vitro*. We identified small molecules that enhanced the transfection efficiency of tumor-associated endothelial cells, but not normal human endothelial cells or cancer cells. IV administration of our targeted, reversibly masked complexes into mice, bearing human pancreatic tumor and endothelial cells, specifically increased transfection to this tumor microenvironment about 200-fold. Efficacy studies using our optimized targeted delivery of a plasmid encoding thrombospondin-1 eliminated tumors completely after five intravenous injections administered once weekly. We plan to use our targeted, reversibly masked delivery system in the clinic to treat metastatic cancer.

Table 1 compares reversible masking versus the cleavable PEG systems. None of the cleavable PEG systems have shown the exceptionally high, 200-fold increased targeted delivery demonstrated by BIVs coated with small molecule B-turn mimics for specific delivery to the target cell and reversible masking to avoid nonspecific uptake described above. Furthermore, we achieve this high level of targeted delivery without the use of peptides that are known to be immunogenic when multimerized on the surface of delivery vehicles and then repeatedly injected iv. In addition, we demonstrated greater efficacy than these other delivery systems, i.e., complete elimination of aggressive, orthotopic pancreatic cancers after 5 iv injections administered once a week. At best, the receptor-targeted nanocomplex (RTN) with endosomally cleavable PEG and RGD integrin-targeting peptide showed only a 2-fold increased delivery to subcutaneous neuroblastoma tumors [11]. Furthermore, only a 75% reduction in tumor size after 7 iv injections administered every 48 h was achieved using this optimized formulation. Targeted delivery systems can also be less efficient in delivery to the target compared to the non-PEGylated, nontargeted formulations as shown for the MEND system (Table 1) [12, 13, 32]. Not surprising, no efficacy in any disease model has been reported for this delivery system. In summary, we have defined and distinguished our novel reversible masking versus PEGylation and demonstrated its superior use for avoiding nonspecific uptake in vivo.

## Figures and Tables

**Table 1:** Comparison of recent, improved targeted delivery systems.

NP* type	Increase to NT**	Decrease to NT***	Efficacy of targeted delivery system
BIVs + small molecules + reversible masking	200-fold	None	Complete elimination of aggressive, orthotopic pancreatic cancers after 5 iv injections administered once a week [9]
RTNs with endosomally cleavable PEG	2-fold	None	75% reduction in subcutaneous neuroblastoma tumor size after 7 iv injections administered every 48 h [10, 11]
MEND + pH-sensitive fusogenic peptide or a cell penetrating peptide + cleavable PEG	None	2-fold	None reported [12, 13]
FRT + cleavable PEG	1-fold	None	None reported [14]
Copolymers + cleavable PEG	None	None	None reported [15]
Liposomes + cell penetrating peptide + cleavable PEG	1.3-fold	None	None reported [16, 17]

*Nanoparticle (NP).

**Nontargeted (NT); increased delivery of the targeted NP to the target compared with the corresponding nontargeted formulation.

***Nontargeted (NT); decreased delivery of the targeted NP to the target compared with the corresponding nontargeted formulation.

## Abbreviations

BIV: Bilamellar invaginated vesicle
NP: Nanoparticle
Iv: Intravenous
RM: Reversible masking.

## References

[1] Templeton NS, Lasic DD, Frederik PM, Strey HH, Roberts DD, Pavlakis GN (1997). Improved DNA: liposome complexes for increased systemic delivery and gene expression. *Nature Biotechnology*.

[2] Templeton NS (2003). Myths concerning the use of cationic liposomes in vivo. *Expert Opinion on Biological Therapy*.

[3] Templeton NS (2009). Nonviral delivery for genomic therapy of cancer. *World Journal of Surgery*.

[4] Templeton NS (2008). *Liposome Gene Transfection*.

[5] Templeton NS (2002). Liposomal delivery of nucleic acids in vivo. *DNA and Cell Biology*.

[6]  Lu C Systemic gene therapy with tumor suppressor TUSC2/FUS1 nanoparticles for recurrent/metastatic lung cancer.

[7] Nemunaitis G, Jay  CM, Maples PB (2011). Hereditary inclusion body myopathy: single patient response to intravenous dosing of *GNE * gene lipoplex. *Human Gene Therapy*.

[8] Nemunaitis G, Maples PB, Jay C (2010). Hereditary inclusion body myopathy: single patient response to *GNE* gene Lipoplex therapy. *Journal of Gene Medicine*.

[9] Shi Q, Nguyen AT, Angell Y (2010). A combinatorial approach for targeted delivery using small molecules and reversible masking to bypass nonspecific uptake in vivo. *Gene Therapy*.

[10] Grosse SM, Tagalakis AD, Mustapa MFM (2010). Tumor-specific gene transfer with receptor-mediated nanocomplexes modified by polyethylene glycol shielding and endosomally cleavable lipid and peptide linkers. *FASEB Journal*.

[11] Tagalakis AD, Grosse SM, Meng QH (2011). Integrin-targeted nanocomplexes for tumour specific delivery and therapy by systemic administration. *Biomaterials*.

[12] Hatakeyama H, Akita H, Harashima H (2011). A multifunctional envelope type nano device (MEND) for gene delivery to tumours based on the EPR effect: a strategy for overcoming the PEG dilemma. *Advanced Drug Delivery Reviews*.

[13] Hatakeyama H, Akita H, Ito E (2011). Systemic delivery of siRNA to tumors using a lipid nanoparticle containing a tumor-specific cleavable PEG-lipid. *Biomaterials*.

[14] McNeeley KM, Karathanasis E, Annapragada AV, Bellamkonda RV (2009). Masking and triggered unmasking of targeting ligands on nanocarriers to improve drug delivery to brain tumors. *Biomaterials*.

[15] Thambi T, Yoon HY, Kim K (2011). Bioreducible block copolymers based on poly(ethylene glycol) and poly(*γ*-benzyl L-glutamate) for intracellular delivery of camptothecin. *Bioconjugate Chemistry*.

[16] Kuai R, Yuan W, Li W (2011). Targeted delivery of cargoes into a murine solid tumor by a cell-penetrating peptide and cleavable poly(ethylene glycol) comodified liposomal delivery system via systemic administration. *Molecular Pharmacology*.

[17] Kuai R, Yuan W, Qin Y (2010). Efficient delivery of payload into tumor cells in a controlled manner by TAT and thiolytic cleavable PEG Co-modified liposomes. *Molecular Pharmaceutics*.

[18] Ramesh R, Saeki T, Smyth Templeton N (2001). Successful treatment of primary and disseminated human lung cancers by systemic delivery of tumor suppressor genes using an improved liposome vector. *Molecular Therapy*.

[19] Shi HY, Liang R, Templeton NS, Zhang M (2002). Inhibition of breast tumor progression by systemic delivery of the maspin gene in a syngeneic tumor model. *Molecular Therapy*.

[20] Tirone TA, Fagan SP, Templeton NS, Wang X, Brunicardi FC (2001). Insulinoma-induced hypoglycemic death in mice is prevented with beta cell-specific gene therapy. *Annals of Surgery*.

[21] Liu SH, Smyth-Templeton N, Davis AR (2011). Multiple treatment cycles of liposome-encapsulated adenoviral RIP-TK gene therapy effectively ablate human pancreatic cancer cells in SCID mice. *Surgery*.

[22] Liu S, Ballian N, Belaguli NS (2008). PDX-1 acts as a potential molecular target for treatment of human pancreatic cancer. *Pancreas*.

[23] Pan WH, Xin P, Morrey JD, Clawson GA (2004). A self-processing ribozyme cassette: utility against human papillomavirus 11 E6/E7 mRNA and hepatitis B virus. *Molecular Therapy*.

[24] Sternberg B (1996). Morphology of cationic liposome/DNA complexes in relation to their chemical composition. *Journal of Liposome Research*.

[25] Felgner PL, Gadek TR, Holm M (1987). Lipofection: a highly efficient, lipid-mediated DNA-transfection procedure.. *Proceedings of the National Academy of Sciences of the United States of America*.

[26] Felgner JH, Kumar R, Sridhar CN (1994). Enhanced gene delivery and mechanism studies with a novel series of cationic lipid formulations. *The Journal of Biological Chemistry*.

[27] Senior J, Delgado C, Fisher D, Tilcock C, Gregoriadis G (1991). Influence of surface hydrophilicity of liposomes on their interaction with plasma protein and clearance from the circulation: studies with poly(ethylene glycol)-coated vesicles. *Biochimica et Biophysica Acta*.

[28] Papahadjopoulos D, Allen TM, Gabizon A (1991). Sterically stabilized liposomes: improvements in pharmacokinetics and antitumor therapeutic efficacy. *Proceedings of the National Academy of Sciences of the United States of America*.

[29] Gabizon A, Catane R, Uziely B (1994). Prolonged circulation time and enhanced accumulation in malignant exudates of doxorubicin encapsulated in polyethylene-glycol coated liposomes. *Cancer Research*.

[30] Gordon KB, Tajuddin A, Guitart J (1995). Hand-foot syndrome associated with liposome-encapsulated doxorubicin therapy. *Cancer*.

[31] Uziely B, Jeffers S, Isacson R (1995). Liposomal doxorubicin: antitumor activity and unique toxicities during two complementary phase I studies. *Journal of Clinical Oncology*.

[32] Sakurai Y, Hatakeyama H, Akita H (2009). Efficient short interference rna delivery to tumor cells using a combination of octaarginine, gala and tumor-specific, cleavable polyethylene glycol system. *Biological and Pharmaceutical Bulletin*.

[33] Gustafsson J, Arvidson G, Karlsson G, Almgren M (1995). Complexes between cationic liposomes and DNA visualized by cryo-TEM. *Biochimica et Biophysica Acta*.

[34] Templeton NS, Lasic DD (1999). New directions in liposome gene delivery. *Applied Biochemistry and Biotechnology—Part B*.

[35] Jain RK (1994). Barriers to drug delivery in solid tumors. *Scientific American*.

[36] Jain RK (1991). Haemodynamic and transport barriers to the treatment of solid tumours. *International Journal of Radiation Biology*.

[37] Jain RK (1999). Transport of molecules, particles, and cells in solid tumors. *Annual Review of Biomedical Engineering*.

[38] Templeton NS Non-viral vectors for the treatment of disease.

[39] Repits J, Sterjovski J, Badia-Martinez D (2008). Primary HIV-1 R5 isolates from end-stage disease display enhanced viral fitness in parallel with increased gp120 net charge. *Virology*.

[40] Cilliers T, Nhlapo J, Coetzer M (2003). The CCR5 and CXCR4 coreceptors are both used by human immunodeficiency virus type 1 primary isolates from subtype C. *Journal of Virology*.

[41] Lee E, Hall RA, Lobigs M (2004). Common E protein determinants for attenuation of glycosaminoglycan-binding variants of Japanese encephalitis and West Nile viruses. *Journal of Virology*.

[42] Markoff L, Falgout B, Chang A (1997). A conserved internal hydrophobic domain mediates the stable membrane integration of the dengue virus capsid protein. *Virology*.

[43] Reeves JD, Schulz TF (1997). The CD4-independent tropism of human immunodeficiency virus type 2 involves several regions of the envelope protein and correlates with a reduced activation threshold for envelope-mediated fusion. *Journal of Virology*.

[44] Andeweg AC, Boers PHM, Osterhaus ADME, Bosch ML (1995). Impact of natural sequence variation in the V2 region of the envelope protein of human immunodeficiency virus type 1 on syncytium induction: a mutational analysis. *Journal of General Virology*.

[45] Behr JP, Demeneix B, Loeffler JP, Perez-Mutul J (1989). Efficient gene transfer into mammalian primary endocrine cells with lipopolyamine-coated DNA. *Proceedings of the National Academy of Sciences of the United States of America*.

[46] Felgner PL, Ringold GM (1989). Cationic liposome-mediated transfection. *Nature*.

[47] Pinnaduwage P, Huang L (1989). The role of protein-linked oligosaccharide in the bilayer stabilization activity of glycophorin A for dioleoylphosphatidylethanolamine liposomes. *Biochimica et Biophysica Acta*.

[48] Leventis R, Silvius JR (1990). Interactions of mammalian cells with lipid dispersions containing novel metabolizable cationic amphiphiles. *Biochimica et Biophysica Acta*.

[49] Rose JK, Buonocore L, Whitt MA (1991). A new cationic liposome reagent mediating nearly quantitative transfection of animal cells. *BioTechniques*.

[50] Loeffler JP, Behr JP (1993). Gene transfer into primary and established mammalian cell lines with lipopolyamine-coated DNA. *Methods in Enzymology*.

[51] Hood JD, Bednarski M, Frausto R (2002). Tumor regression by targeted gene delivery to the neovasculature. *Science*.

